# Integrating information retrieval with distant supervision for Gene Ontology annotation

**DOI:** 10.1093/database/bau087

**Published:** 2014-09-01

**Authors:** Dongqing Zhu, Dingcheng Li, Ben Carterette, Hongfang Liu

**Affiliations:** ^1^Department of Health Sciences Research, Mayo Clinic, 200 First St SW, Rochester, MN 55905 and ^2^Department of Computer & Information Sciences, University of Delaware, 101 SMITH HALL, Newark, DE 19716, USA

## Abstract

This article describes our participation of the Gene Ontology Curation task (GO task) in BioCreative IV where we participated in both subtasks: A) identification of GO evidence sentences (GOESs) for relevant genes in full-text articles and B) prediction of GO terms for relevant genes in full-text articles. For subtask A, we trained a logistic regression model to detect GOES based on annotations in the training data supplemented with more noisy negatives from an external resource. Then, a greedy approach was applied to associate genes with sentences. For subtask B, we designed two types of systems: (i) search-based systems, which predict GO terms based on existing annotations for GOESs that are of different textual granularities (i.e., full-text articles, abstracts, and sentences) using state-of-the-art information retrieval techniques (i.e., a novel application of the idea of distant supervision) and (ii) a similarity-based system, which assigns GO terms based on the distance between words in sentences and GO terms/synonyms. Our best performing system for subtask A achieves an F1 score of 0.27 based on exact match and 0.387 allowing relaxed overlap match. Our best performing system for subtask B, a search-based system, achieves an F1 score of 0.075 based on exact match and 0.301 considering hierarchical matches. Our search-based systems for subtask B significantly outperformed the similarity-based system.

**Database URL:** https://github.com/noname2020/Bioc

## Introduction

The Gene Ontology (GO) provides a set of concepts for annotating functional descriptions of genes and proteins in biomedical literature. The resulting annotated databases are useful for large-scale analysis of gene products. However, performing GO annotation requires expertise from well-trained human curators. Owing to the fast expansion of biomedical data, GO annotation becomes extremely labor-intensive and costly. Thus, texting mining tools that can assist GO annotation and reduce human effort are highly desired (1–3).

To promote research and tool development for assisting GO curation from biomedical literature, the Critical Assessment of Information Extraction in Biology (BioCreative) IV organized Gene Ontology Curation task (GO task) in 2013 (4). There are two subtasks: A) identification of GO evidence sentences (GOESs) for relevant genes in full-text articles and B) prediction of GO terms for relevant genes in full-text articles. The training set of GO task contains 100 full-text journal articles [in BioC format (5)], while the development and test sets each have 50 articles. Task organizers also provided ground truth annotations for the training and development sets to all participants (5). Table 1 gives the detailed statistics about genes, gene-related passages and GO terms in the GO task data.

**Table 1.** Statistics of the data set for BioCreative IV Track 4 GO Task

GO task data	Training set	Development set	Test set
Number of full-text articles	100	50	50
Number of genes	300	171	194
Number of gene-associated passages	2234	1247	1681
Number of GO terms	954	575	644

The following shows two sample passages and the corresponding key information in the training and development sets:

**Key information for sample passage 1:**
<infon key=“**gene**”>cdc-14(173945)</infon>
<infon key=“**go-term**”>embryo development ending in birth or egg hatching|GO:0009792</infon>
<infon key=“**goevidence**”>IMP</infon>
<text>However, of all components tested, only the depletion of the *C. elegans* homologue of the budding yeast Cdc14p phosphatase caused embryonic lethality in the offspring of injected worms (Table 1).</text>

**Key information for sample passage 2:**
<infon key=“**gene**”>cdc-14(173945)</infon>
<infon key=“**go-term**”>phosphatase activity|GO: 0016 791</infon>
<infon key=“**goevidence**”>NONE</infon>
<text>CeCDC-14 is a phosphatase and localizes to the central spindle and the midbody</text>

Given a set of relevant genes, for subtask A, we need to find GOESs, while for subtask B, we need to assign GO terms to each article (primarily based on the gene-related sentences identified in subtask A).

In this article, we describe our participation systems for the GO task. For subtask A, we trained a logistic regression (LR) model to detect GOESs using the training data supplemented with noisy negatives from an external resource. A greedy approach was applied to associate relevant genes with sentences. For subtask B, we designed two types of systems: (i) search-based systems, which predict GO terms based on existing annotations for GOESs that are of different textual granularities (i.e., full-text articles, abstracts, and sentences) using state-of-the-art information retrieval techniques and (ii) a similarity-based system, which assigns GO terms based on the distance between words in sentences and GO terms/synonyms.

In the following sections, we will first describe our systems in more detail. Then, we will present and discuss the official evaluation results. Finally, we draw conclusion and point possible directions for future work.

## System description

### Subtask A

In this subtask, given a full-text article, we need to identify GOESs and associate genes related to these sentences. The task can be defined as a supervised machine learning task by considering GOESs as positives and all other sentences as negatives. As positives and negatives are from the same pool of articles, the resultant models may be overfitted. We supplemented negatives with unlabeled excerpts from GeneRIF (6) records aiming for better models based on our prior experience on distant supervision, i.e. use existing resources to obtain weakly labeled instances for training machine learning classifiers (7, 8).

#### Data preprocessing

We extract positive and negative instances (i.e. sentences) from both training and developing sets to train our model. The training set contains 1318 positive and 26 868 negative instances, while the development set gives 558 positive and 14 580 negative sentences.

We use GeneRIF as an unlabeled data pool, which contains excerpts from literature about the functional annotation of genes described in EntrezGene. In particular, each record contains a taxonomy ID, a Gene ID, a PMID and a GeneRIF text excerpt extracted from literature. We randomly obtain 20 000 excerpts from human GeneRIF records with at most two records per Gene ID and the corresponding articles not associated with any GO annotations (GOA) record based on GOA information available in iProClass (9). We assume those excerpts have a higher chance to be negatives, assuming that if the excerpts are evidence excerpts, the corresponding article has a higher probability to be included in GOA. The rationale behind this assumption is that the scope of the functional annotation in GeneRIF is broader than that of GO. Besides the scope of GO annotation, GeneRIF also includes phenotypic and disease information that are not the subject of GO annotation. Note that this assumption does not guarantee all excerpts obtained to be true negatives.

#### Feature extractions

##### Bag-of-word (BOW) features

For each sentence, we generate a vector of stemmed words.

##### Bigram features

For each sentence, we generate a vector of bigrams by concatenating every two neighboring stemmed words in the sentence. We also have two boundary bigrams (SOS_Lw and Rw_EOS) where SOS indicates ‘Start of the Sentence’, EOS indicates ‘End of the Sentence’, Lw, the leftmost stemmed word and Rw, the rightmost stemmed word.

##### Section feature

For each sentence, we include a feature to indicate which section the sentence is from (i.e. title, abstract, introduction, methods, discussion, etc.).

##### Topic features

These features are generated by Latent Dirichlet Allocations (10), which can effectively group similar instances together based on their topics (11–14).

##### Presence of relevant genes

Because relevant genes of each article have been provided, we also use dictionary lookup to check the presence of relevant genes in the sentence.

#### Model training

We apply LR to predict labels for each instance. In particular, we impose a constraint on model parameters in a regularized LR to avoid overfitting and to improve the prediction performance on unseen instances. Note that LR assigns probability scores to each class. In a task with skewed class distribution, a threshold can be chosen to optimize the performance.

#### Assembling gene and evidence sentence pairs

For each article, all relevant genes are provided. Therefore, we use a greedy approach to associate evidence excerpts with the relevant genes. The approach includes four steps:

##### Step 1. Direct matching with dictionary lookup

Direct dictionary lookup is done for each predicted positive sentence to detect whether there are relevant genes appearing in the sentence. If so, the corresponding genes found are assigned to that sentence.

##### Step 2. Family name inferred

Because genes belonging to the same family can appear as plurals in the document, we assemble a dictionary of family names based on the gene mentions provided. For each mention of the family name in a sentence (using direct string matching), all of the members of that family in the gene list are assigned to the sentence.

##### Step 3. Gene assignment based on proximity

For the remaining predicted positive sentences with no relevant gene mentioned, we assume that prior sentences would contain the gene information. For positive sentence S, we perform direct string matching using the gene list provided and the family name dictionary assembled in Step 2 on all prior sentences belonging to the same section of S. Gene hits are identified similarly as in Steps 1 and 2. We then assign gene hits from the closest one (among all prior sentences with gene hits) to S.

##### Step 4. Assignment based on gene-sentence distributions

For genes failed to be associated with any predicted positive sentence, we picked sentences containing the corresponding genes with the largest positive probability score (assigned by the LR model) to be the evidence sentences.

#### Submissions for subtask A

We used LR-TRIRLS (15), which implements ridge regression, to build LR models. We chose a threshold of 0.1 based on the performance of the model trained using the training set and evaluated using the development set, where if a sentence has a probability >0.1 to be positive, then we consider it as positive. We submitted three runs (A1, A2 and A3) for subtask A. Runs A1 and A2 used different sets of unlabeled instances sampled from GeneRIF, and Run A3 combined the results from A1 and A2.

### Subtask B

In this section, we describe two systems that generated the first two runs of Task B. The basic idea is to leverage existing GOA to label new articles. In particular, we search for relevant documents (sentences, abstracts or full-text articles) that have existing GOA to the target article, and then score and aggregate these existing GOA to produce the GOA for the target article.

#### System B1

**Figure 1.**
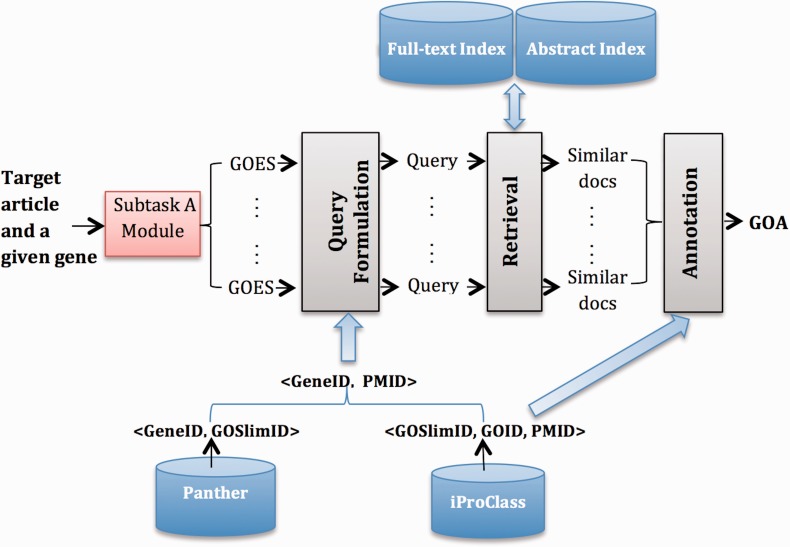
Overview of System B1.

Figure 1 gives an overview of System B1. We highlight external resources in blue and system modules with gray. Next, we describe each part in detail.

##### Resources

We use the following external resources: (i) Panther (16), from which we build <GeneID, GOSlimID> pairs; (ii) iProClass (9), from which we obtain <GOSlimID, GOID, PMID> triplets; (iii) a collection of PMC full-text articles that serve as the source for finding relevant documents and (iv) a collection of PubMed abstracts, used as a complementary source for retrieving, because for some GOA records, only abstracts are publically available for the corresponding articles.

##### Retrieval

We build indexes for the abstract collection and the full-text collection, respectively, using the Indri (17) search engine. In particular, we use the Porter stemmer for stemming words in the documents. We choose the query likelihood language model as our retrieval model. This model scores documents for queries as a function of the probability that query terms would be sampled (independently) from a bag containing all the words in that document. Formally, the scoring function is a sum of the logarithms of smoothed probabilities:

$$\text{score}\left(D,Q\right)=\log P\left(Q|D\right)=\overset{n}{\underset{i=1}{\sum }}\log\frac{tf_{q_{i},D}+\mu \frac{tf_{q_{i},C}}{\left(\left|C\right|\right)}}{\left|D\right|+\mu }$$

where $q_{i}$ is the i^th^ query term, |D| and |C| are the document and collection lengths in words, respectively, $tf_{q_{i},D}$ and $tf_{q_{i},C}$ are the document and collection term frequencies of $q_{i}$, respectively, and μ is the Dirichlet smoothing parameter. The Indri retrieval engine supports this model by default.

##### Query formulation

We formulate a query for each detected GOES from the output of subtask A. In particular, we filter stop words in the sentence using a standard stop word list. We leverage information in <GeneID, GOSLIM, GO> triples to reduce the GO candidate list (denoted as **C**), and then build a PMID candidate list by incorporating information in the <PMID, GOA> pairs. The following lists the detailed steps:

- Given a gene G, we have a list of <G, GOES> pairs.
- For each <G, GOES> pair, we find the corresponding <G, GOSlimID> pairs.
- For each <G, GOSlimID> pair, we get a list of PMIDs based on <GOSlimID, GOID, PMID> triplets.
- Combine all PMIDs for G to get a <G, L> pair, where L is the PMID candidate list (a reduced searching list) for G.

##### Annotation

The output from the retrieval model for a given <GeneID, GOES> pair is a list of documents ranked by their relevance scores. Based on the <GOSlimID, GOID, PMID> triplets, we obtain GOIDs for top-ranked k documents, and then weight each GOID by their corresponding document relevance score. We further aggregate scores of each GOID and take the top-ranked m GOID for each GOES. Finally, we combine GOID across all GOES, rank them according to their occurrences and keep GOID, which occurs more than ptimes. For our submission, we set <k, m, p > to <7, 10, 4> by training them on the 150 articles (i.e. the combination of training and development sets).

#### System B2

**Figure 2.**
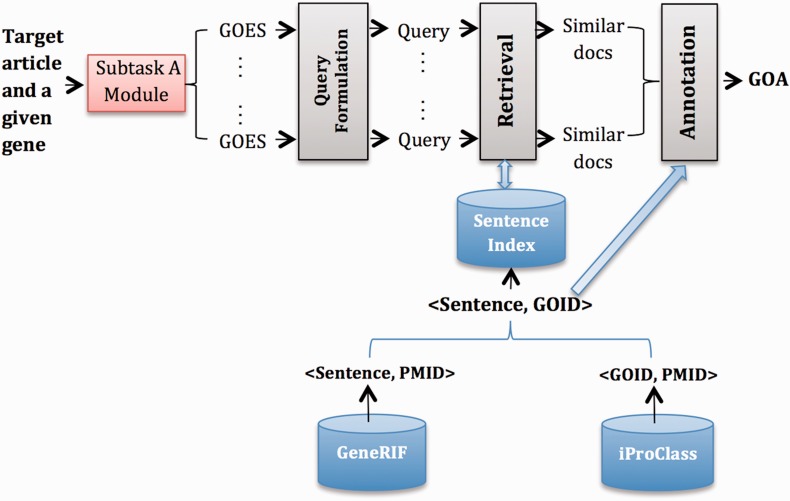
Overview of System B2.

Figure 2 gives an overview of System B2, which has similar modules to System B1. The major difference is that we use GeneRIF (6) as the external resource. In particular, we extract <Sentence, GOID> pairs from GeneRIF where the corresponding articles are cited as evidence of GOA records in iProClass and built an index for this collection of sentences. Therefore, the output from the Retrieval model is a ranked list of sentences, which we further converted to a ranked list of GOID based on <Sentence, GOID> pairs. Finally, in the Annotation module, we did the following:

1. Starting from an initial list that contains top-ranked k GOID, select GOID one by one down the list until the score difference of current GOID with the topmost GOID is above threshold h.
2. Aggregate GOID frequency across all GOES associated with a particular gene, and rank GOID by frequency.
3. Take the top-ranked m GOID for each gene.

#### Submissions for subtask B.

System B1 and System B2 were implemented in Indri (18). By training them on the 150 articles (i.e. the combination of training and development sets), we set < k, m, p > to <7, 10, 4> for System B1 and <k, h, m> to <5, 0.1, 3> for System B2. We submitted three runs (B1, B2 and B3) where Run B1 is the output of System B1 and Run B2 is the output of System B2. Run B3 is the output of a string matching algorithm. Specifically, we obtained all words in the sentences that are aligned to GO terms and synonyms when ignoring lexical variations (System B3). We then computed the Jaccard distance (19) between those matched words with GO terms and synonyms. A threshold of 0.75 was used for GO term assignment.

## Evaluation metrics

Both subtasks are evaluated using the standard precision (P), recall (R) and F1-measure (F1) scores (4). However, there are two different criteria for determining a match between a candidate sentence and the ground truth sentence: (i) exact match between sentence boundaries and (ii) partial overlapping. Subtask B is also evaluated by P, R and F1 based on two different matching criteria: flat or hierarchical. For the flat P, R and F1, a match occurs when the predicted GO term is exactly the same as the gold standard. For hierarchical P, R and F1, a match occurs when the predicted GO term has a common ancestor with the ground truth GO term.

## Results and discussion

Table 2 presents the official evaluation results of subtask A. Runs A1 and A3 obtain comparable F1 scores. Run A2 has a lower F1 score because of the relatively low performance for recall. Note that the performance difference between Runs A1 and A2 was purely because of different noisy negatives sampled from GeneRIF.

**Table 2.** Official evaluation results for subtask A using traditional P, R and F-Measure (F1)

System	Overlap match			Exact match		
	P	R	F1	P	R	F1
A1	0.313	0.503	0.386	0.219	0.352	0.270
A2	0.314	0.442	0.367	0.220	0.310	0.257
A3	0.307	0.524	0.387	0.214	0.366	0.270

Both strict exact match and relaxed overlap measure are considered.

During the development phase of systems for subtask A, we assessed the performance with or without the use of additional GeneRIF excerpts and the contributions of individual types of features. We found that the use of an unlabeled data set sampled from GeneRIF improved the F1 score by 0.03 compared with the baseline, which uses only positives and negatives from the training data set and BOW features. Also, including other features (bigrams, gene existence, section and topic features) led to performance improvement over the baseline. In particular, section feature improved the F1 score by 0.01. Bigram and gene presence features each brought an improvement of 0.008. Topic features further added 0.003 when the number of topics was set to 100.

Table 3 presents the official evaluation results of subtask B. The exact F1 scores for both types of systems are <0.1. System B1 achieves 0.301 for Hierarchical-F1. Our search-based systems (i.e. B1 and B2) outperformed the similarity-based systems (i.e. B3) significantly.

**Table 3.** Official evaluation results for subtask B using traditional (flat) precision (P), recall (R) and F1-measure (F1) and hierarchical precision (hP), recall (hR) and F1-measure (hF1)

System	Flat			Hierarchical		
	P	R	F1	hP	hR	hF1
B1	0.054	0.149	0.079	0.243	0.459	0.318
B2	0.088	0.076	0.082	0.250	0.263	0.256
B3	0.029	0.039	0.033	0.196	0.310	0.240

We were not aware of the need of containing experimental methods for detecting GO evidence excerpts and assigning GO terms as specified by the annotation guideline. This may explain why the use of section features in subtask A has the most gain in the F1 score. Additionally, we sampled only from human GeneRIF records with at most two records per gene. The rationale behind it is to avoid overrepresentation of popular studied genes and their homologous genes. It is not clear whether such sampling approach has impact on the performance of the system.

Note that the use of GOSlim in System B1 aims to reduce the number of candidate GO terms for consideration. As subtask B depends on subtask A, it is not clear how well our search-based methods for subtask B can achieve giving the gold standard output from subtask A. Owing to the time constraint, we leave this interesting investigation as a potential future investigation.

## Conclusion and future work

Through the participation of the GO task, we investigated the use of distant supervision for detecting sentences for GO annotation assignment and explored the use of information retrieval techniques for finding relevant existing GOA and used them for assigning GO terms to new articles.

The results look promising compared with previous challenges. However, there is still much room for improvement. Specifically, we plan to explore advanced text modeling methods including deep learning (20–23) and hierarchical/supervised topic modeling (24–26) for the task. We can make use of unlabeled text for feature extractions or build deep belief networks for sparse feature learning. With enough GOA, we can explore the use of hierarchical/supervised topic modeling for predicting GOA given evidence sentences.

## Funding

The work was supported by US National Science Foundation (ABI: 0845523) and US National Institute of Health (R01LM009959). Funding for open access charge: US NSF (ABI:0845523) and US NIH (R01LM009959).

*Conflict of interest*. None declared.
